# Supplementary material for: Effectiveness of the capability approach in rehabilitation for persons with neuromuscular diseases: A controlled before-after study
Source: PLoS One. 2025 Sep 23;20(9):e0332388. doi: 10.1371/journal.pone.0332388 (PMC12456807; doi:10.1371/journal.pone.0332388)
Supplement: S1 Table — USER-P: Utrecht Scale for Evaluation of Rehabilitation Participation. (DOCX) [file pone.0332388.s001.docx]

**S1 Table. Means and standard deviations on the USER-P for both groups at baseline and follow-up.**

|  |  | **Baseline (T0)** | | | **6-month follow-up (T1)** | | |
| --- | --- | --- | --- | --- | --- | --- | --- |
|  |  | **N** | **Mean** | **SD** | **N** | **Mean** | **SD** |
| **USER-P frequency** | **Usual care** | 29 | 31.1 | 10.5 | 27 | 31.1 | 10.7 |
|  | **Capability care** | 27 | 33.0 | 9.2 | 28 | 32.4 | 9.8 |
| **USER-P restrictions** | **Usual care** | 29 | 73.6 | 15.0 | 27 | 73.4 | 15.5 |
|  | **Capability care** | 26 | 75.7 | 18.4 | 27 | 75.7 | 17.9 |
| **USER-P satisfaction** | **Usual care** | 29 | 62.5 | 14.1 | 27 | 64.3 | 16.1 |
|  | **Capability care** | 27 | 59.3 | 15.9 | 28 | 60.0 | 12.8 |

USER-P: Utrecht Scale for Evaluation of Rehabilitation Participation
