# Supplementary material for: Effectiveness of the capability approach in rehabilitation for persons with neuromuscular diseases: A controlled before-after study
Source: PLoS One. 2025 Sep 23;20(9):e0332388. doi: 10.1371/journal.pone.0332388 (PMC12456807; doi:10.1371/journal.pone.0332388)
Supplement: S2 Table — EQ5D-5L: EuroQol-5D-5L; VAS: visual analogue scale; LSS: level sum score. (DOCX) [file pone.0332388.s002.docx]

**S2 Table. Means and standard deviations on the EQ-5D-5L for both groups at baseline and follow-up.**

|  |  | **Baseline (T0)** | | | **6-month follow-up (T1)** | | |
| --- | --- | --- | --- | --- | --- | --- | --- |
|  |  | **N** | **Mean** | **SD** | **N** | **Mean** | **SD** |
| **EQ-5D-5L VAS** | **Usual care** | 29 | 61.6 | 15.5 | 27 | 66.5 | 14.6 |
|  | **Capability care** | 27 | 59.7 | 17.9 | 28 | 61.9 | 12.9 |
| **EQ-5D-5L LSS** | **Usual care** | 29 | 12.4 | 3.3 | 27 | 12.2 | 3.5 |
|  | **Capability care** | 27 | 10.8 | 3.5 | 28 | 11.1 | 3.0 |

EQ-5D-5L: EuroQol-5D-5L; VAS: visual analogue scale; LSS: level sum score
