# Supplementary material for: Effectiveness of the capability approach in rehabilitation for persons with neuromuscular diseases: A controlled before-after study
Source: PLoS One. 2025 Sep 23;20(9):e0332388. doi: 10.1371/journal.pone.0332388 (PMC12456807; doi:10.1371/journal.pone.0332388)
Supplement: S3 Table — ICECAP-A: ICEpop CAPability measure for Adults. (DOCX) [file pone.0332388.s003.docx]

**S3 Table. Means and standard deviations on the ICECAP-A for both groups at baseline and follow-up.**

|  |  | **Baseline (T0)** | | | **6-month follow-up (T1)** | | |
| --- | --- | --- | --- | --- | --- | --- | --- |
|  |  | **N** | **Mean** | **SD** | **N** | **Mean** | **SD** |
| **ICECAP-A sum score** | **Usual care** | 29 | 15.2 | 2.3 | 28 | 14.9 | 2.3 |
|  | **Capability care** | 27 | 14.5 | 1.9 | 28 | 14.6 | 2.1 |
| **ICECAP-A tariff value** | **Usual care** | 29 | 0.81 | 0.13 | 28 | 0.80 | 0.14 |
|  | **Capability care** | 27 | 0.78 | 0.12 | 28 | 0.79 | 0.13 |

ICECAP-A: ICEpop CAPability measure for Adults
