# Supplementary material for: Effectiveness of the capability approach in rehabilitation for persons with neuromuscular diseases: A controlled before-after study
Source: PLoS One. 2025 Sep 23;20(9):e0332388. doi: 10.1371/journal.pone.0332388 (PMC12456807; doi:10.1371/journal.pone.0332388)
Supplement: S4 Table — SF-36: Medical Outcome Study Short Form-36; PF: physical functioning; RP: role limitations due to physical health problems; RE: role limitations due to emotional health problems; VT: vitality; MH: mental health; SF: social functioning; BP: bodily pain; GH: general health. (DOCX) [file pone.0332388.s004.docx]

**S4 Table. Means and standard deviations on the SF-36 for both groups at baseline and follow-up.**

|  |  | **Baseline (T0)** | | | **6-month follow-up (T1)** | | |
| --- | --- | --- | --- | --- | --- | --- | --- |
|  |  | **N** | **Mean** | **SD** | **N** | **Mean** | **SD** |
| **SF-36 PF** | **Usual care** | 29 | 43.8 | 26.3 | 27 | 40.2 | 25.9 |
|  | **Capability care** | 27 | 49.6 | 26.1 | 28 | 48.9 | 26.3 |
| **SF-36 RP** | **Usual care** | 29 | 46.1 | 23.8 | 27 | 43.8 | 21.2 |
|  | **Capability care** | 27 | 53.5 | 28.1 | 28 | 54.5 | 22.9 |
| **SF-36 RE** | **Usual care** | 29 | 66.4 | 29.9 | 27 | 67.6 | 31.5 |
|  | **Capability care** | 27 | 68.8 | 26.3 | 28 | 69.9 | 23.7 |
| **SF-36 VT** | **Usual care** | 29 | 47.0 | 16.3 | 27 | 45.8 | 17.7 |
|  | **Capability care** | 27 | 47.2 | 15.5 | 28 | 47.5 | 15.5 |
| **SF-36 MH** | **Usual care** | 29 | 70.3 | 21.1 | 27 | 71.5 | 18.9 |
|  | **Capability care** | 27 | 72.4 | 15.5 | 28 | 72.5 | 15.0 |
| **SF-36 SF** | **Usual care** | 29 | 68.5 | 25.6 | 27 | 67.6 | 23.8 |
|  | **Capability care** | 27 | 71.8 | 20.1 | 28 | 69.6 | 20.2 |
| **SF-36 BP** | **Usual care** | 29 | 56.7 | 21.8 | 27 | 60.0 | 22.1 |
|  | **Capability care** | 27 | 65.0 | 25.7 | 28 | 63.2 | 20.0 |
| **SF-36 GH** | **Usual care** | 29 | 41.2 | 18.7 | 27 | 43.3 | 20.9 |
|  | **Capability care** | 27 | 41.3 | 17.1 | 28 | 39.2 | 19.0 |

SF-36: Medical Outcome Study Short Form-36; PF: physical functioning; RP: role limitations due to physical health problems; RE: role limitations due to emotional health problems; VT: vitality; MH: mental health; SF: social functioning; BP: bodily pain; GH: general health
