# Supplementary material for: Effectiveness of the capability approach in rehabilitation for persons with neuromuscular diseases: A controlled before-after study
Source: PLoS One. 2025 Sep 23;20(9):e0332388. doi: 10.1371/journal.pone.0332388 (PMC12456807; doi:10.1371/journal.pone.0332388)
Supplement: S5 Table — USER-P: Utrecht Scale for Evaluation of Rehabilitation Participation; EQ5D-5L: EuroQol-5D-5L; VAS: visual analogue scale; LSS: level sum score; ICECAP-A: ICEpop CAPability measure for Adults; SF-36: Medical Outcome Study Short Form-36; PF: physical functioning; RP: role limitations due to physical health problems; RE: role limitations due to emotional health problems; VT: vitality; MH: mental health; SF: social functioning; BP: bodily pain; GH: general health. (DOCX) [file pone.0332388.s005.docx]

**S5 Table. Significant covariates for the ANCOVA on secondary outcome measures.**

|  | **Significant p-values per covariate** | | | |
| --- | --- | --- | --- | --- |
|  | **Baseline (T0) score** | **Age** | **Sex** | **Type of NMD** |
| **USER-P frequency** | <0.001 |  |  |  |
| **USER-P restrictions** | <0.001 |  |  | 0.035 |
| **USER-P satisfaction** | <0.001 |  | 0.043 | 0.020 |
| **EQ-5D-5L VAS** | <0.001 |  |  |  |
| **EQ-5D-5L LSS** | <0.001 |  |  |  |
| **ICECAP-A sum score** | <0.001 |  |  | 0.018 |
| **ICECAP-A tariff value** | <0.001 | 0.022 |  | 0.032 |
| **SF-36 PF** | <0.001 |  |  |  |
| **SF-36 RP** | <0.001 |  |  | 0.049 |
| **SF-36 RE** | <0.001 |  |  |  |
| **SF-36 VT** | <0.001 |  |  |  |
| **SF-36 MH** | <0.001 |  |  |  |
| **SF-36 SF** | <0.001 |  |  |  |
| **SF-36 BP** | <0.001 |  |  |  |
| **SF-36 GH** | <0.001 |  | 0.024 |  |

USER-P: Utrecht Scale for Evaluation of Rehabilitation Participation; EQ5D-5L: EuroQol-5D-5L; VAS: visual analogue scale; LSS: level sum score; ICECAP-A: ICEpop CAPability measure for Adults; SF-36: Medical Outcome Study Short Form-36; PF: physical functioning; RP: role limitations due to physical health problems; RE: role limitations due to emotional health problems; VT: vitality; MH: mental health; SF: social functioning; BP: bodily pain; GH: general health
