# Supplementary material for: Effectiveness of the capability approach in rehabilitation for persons with neuromuscular diseases: A controlled before-after study
Source: PLoS One. 2025 Sep 23;20(9):e0332388. doi: 10.1371/journal.pone.0332388 (PMC12456807; doi:10.1371/journal.pone.0332388)
Supplement: Attachment — Submitted filename: Cover letter revision - response to reviewers.docx [file pone.0332388.s009.docx]

To: Giorgia Coratti, Guest Editor PLOS ONE

Nijmegen, 28^th^ of January 2025

Concerning: PONE-D-24-16662

Dear Giorgia Coratti,

Thank you for your e-mail regarding our submitted manuscript *Effectiveness of the capability approach in rehabilitation for persons with neuromuscular diseases: a controlled before-after study.*

We would like to thank the reviewers for their comments. Their feedback clearly improved our manuscript. Below, we will respond to each point addressed by the reviewers and indicate where in the revised version of the manuscript our responses are implemented (line and page numbers refer to the clean copy version of the revised manuscript).

- ***Reviewer #1****: The authors developed an interesting and contemporary study which will support the SMA community. Multidisciplinary care and personalized approaches are key to focus on patients' needs but indeed a personalized/individualized rehabilitation needs a personalized/individualized outcome measure like a GAS for example. Patient's well-being within rehabilitation and more broader within treatment in general, usually also depend on their expectations, which are very individual and usually depending on baseline functional level. I did not see the mention of expectations within this study and I believe that this could also have an impact on the outcome. Patients subgroup analyzes according to functional level or similar baseline criteria (COPM and other scales) could be interesting as well.
  In addition, the training of HCPs outside of the rehabilitation outpatient clinic seems to be a key point that is indeed missing in order to implement adequately the concept.
  Finally, not only a longer follow-up should be taken into consideration but also adding at least a second "day of advice" over time as mentioned in the conclusion.*

We agree with the reviewers valuable comment that personalised rehabilitation needs a personalised outcome measure and that patients’ expectations can have an influence on the impact of rehabilitation on their well-being. At the start of the study we chose a number of outcome measures, with the COPM as primary outcome measure as this is an individualised measure that focuses on the topics identified by the patient as important for them, and has been shown to be a valid measure for evaluating the impact of rehabilitation. As secondary outcome measures, we have chosen to use several measures that are able to measure quality of life and/or capabilities, because we wanted to be able to capture a broad range of impacts on different aspects of well-being. This aligns with the broad conceptualization of well-being in the capability approach. In the discussion (lines 291-335; pages 14-16) we reflect on these outcome measures and whether they were able to capture the impact on well-being in terms of capabilities. Although we agree that patients’ expectations can have an impact on well-being, we did not select a measure that captures patients’ expectations and therefore cannot include this in the analysis; we have added lines 323-330 (page 16) in the discussion section of the manuscript to reflect on this: “In addition, we did not include measures that included persons’ expectations or level of functioning at start of rehabilitation. Although they might have an impact on the outcome of rehabilitation and on a person’s well-being, we expected this to be similar for both groups. In the capability care group, however, we did expect a higher capability well-being, due to the fact that capability care could identify and address broader aspects that impact on well-being. Capability care may therefore have changed a person’s expectations or aspirations in a different way than usual care; however, participants were blinded for which treatment (usual care or capability care) they were receiving. A forthcoming process analysis might provide more insight into this topic.”

Concerning the reviewers next point; due to study setup, it was not possible to train healthcare professionals outside of our clinic, and a longer follow-up was also not possible. A second day of advice is already part of our standard care, as patients have this day of advice yearly (added in line 100, page 6). However, in the current study the follow-up period was only 6 months. Subgroup analyses could definitely be interesting and will be considered for further analysis. More insight into the current results will follow from a forthcoming process analysis, combining quantitative with qualitative data; which is mentioned in our discussion and conclusions (lines 329-330 (page 16), 379-384 (page 18), 398-399 (page 19)). Additionally, a mixed-methods analysis comparing the COPM, ICECAP-A and participant interviews has been performed (manuscript submitted) to provide more insight into the content validity and responsiveness of these measures in the context of rehabilitation for persons with NMD (lines 314-316, page 15).

- ***Reviewer #2****: The authors present results from a study evaluating capability care to usual care among persons with neuromuscular diseases. The authors found changes within groups on several outcomes, but no difference between the groups. The manuscript will be strengthened if the authors consider the following points:

  1. If authors report one-sided p-values, they need to state the direction of the hypothesis and report the corresponding p-value, not pick the significant one-sided p-value to report. In particular, authors report a significant one-sided p-value for ICECAP-A, but then state it is in the direction opposite of what they hypothesized, in which case, the corresponding p-value for their actual hypothesis would not be significant. If authors had wanted to consider an improvement or deterioration, they should report the 2-sided p-value.*

Thank you for these valuable comments concerning the statistical analyses. We have discussed this with our statistician, and we agree that we should report the p-value for our actual hypothesis. As stated in our introduction, lines 79-82 (page 5), we hypothesized that rehabilitation, whether usual or capability care, will improve occupational performance and capabilities of participants. Therefore, we tested whether outcome measures show a significant improvement between T1 and T0. We have made this hypothesis more explicit in our description of the one-sided t-tests, lines 201-202 (page 10). Additionally, because we are conducting one-sided t-tests, our statistician also pointed out that we should use a significance level of 0.025 instead of 0.05 to show a significant effect (line 201, page 10), as this is more in line with what is done in the medical literature.

We have now reported the p-values of all the one-sided t-tests in the direction of our hypothesis, and used the significance level of 0.025. For the primary outcome measure, COPM performance and satisfaction scores, we had already reported the correct p-values and the results are also significant for a significance level of 0.025. Looking at the correct p-values and significance levels for the ICECAP-A implies that there are no significant improvements between T1 and T0 for both groups (usual care and capability care). We have therefore deleted the text and table 4 (after line 275, page 14) where we wrongly described that there was a significant deterioration in ICECAP-A scores in the usual care group. Although we expected a significant improvement, which was not shown, the current results are more in line with our expectations as there is also no significant deterioration found on the ICECAP-A in both groups.

*2. Authors need to further justify why they did not include covariates in the analyses for the secondary outcomes. Just because covariates were not significant for primary outcomes does not guarantee a lack of significance with the secondary outcomes.*

We have discussed this feedback with our statistician, and she agrees with the reviewer to include the covariates in the analyses for the secondary outcome measures. Therefore, we have included the covariates in these analyses and changed the text in the methods (line 204, page 10) and results (lines 266-272, page 13) and have added a table showing the significant covariates in the appendix (Table 8). Including these covariates did not change the outcome of the analyses we were interested in: group still showed no significant effect for all subscales of all secondary outcome measures. This means that there is no significant difference between the usual care and capability care group.

*3. Why are the reported degrees of freedom for COPM-P and COPM-S different (lines 247, 248) when Table 3 says the sample sizes are the same and the same covariates are included in the models?*

We thank the reviewer for pointing out this omission. We have corrected the values in the text (lines 252-254, page 12 in the revised manuscript) as we had made a mistake in reporting the correct values. The correction of the values does not change the significance of the test results.

*4. Table 4 and Table 7 (in the appendix) are both for ICECAP-A, but the information in the Usual Care rows is different. It is not clear why that is and if authors have the data on the full 29 in the Usual Care group, why that isn't reported in the main paper.*

The differences between tables 4 and 7 (of the originally submitted article) are that in table 4 only the participants were included that have completed both T0 and T1 measures and therefore were included in the t-test. Table 7 provides data for the full group of participants; therefore the number of participants differs between T0 en T1. For readability of the article, we chose to report the means and standard deviations of the secondary outcome measures in the appendix. Table 4 was reported in the main text illustrating a significant result; however after considering reviewers feedback point 1 we have changed this (the result was not significant) and deleted table 4 from the main text (see response to point 1). Table 7 (now Table 6) is still included in the appendix with correct values.

*Minor points:

1. Authors should state the number of participants and time between assessments in the abstract.*

We have adapted the text (lines 30-31, page 2) to include number of participants and time between assessments in the abstract.

*2. line 111: "is was determined" should be "it was determined"*

We have adapted the text in line 111 (line 112, page 6 in revised manuscript).

*3. In Table 2, authors should include the standard deviation for age*

We have included standard deviation for age in Table 2.

- **Reviewer #3**: This is a well designed and written paper that contributes to the understanding of the impact of rehabilitation in NMD.
  I would recommend to expand the explanation on the intervention provided. As it's currently presented the study, could not be replicated by others.

Thank you for your kind words. For readability of the current article, we chose not to describe the capability care intervention in detail. We have added some brief information about the intervention (lines 127-131, page 7), and refer to the article that describes both the intervention and its development in detail (lines 131-132, page 7, reference 13). Due to the fact that it is an individualised intervention developed for a specific setting, it would be hard to replicate the exact intervention. For replication the intervention would need to be tailored to the specific setting; therefore we have chosen to describe the development of the intervention, including choices we made during this development, in detail in a separate paper (reference 13), so that others can adapt the intervention to the needs of their setting.

Regarding data availability, we have now stored our data in in a Data Acquisition Collection (DAC) in the Radboud Data Repository (https://doi.org/10.34973/c7cr-0z09) to which access can only be obtained after being invited by the first author (EP) and signing a contract. We therefore have adapted the text in lines 437-439, page 21.

We hope that our responses are clear and that you will accept our revision of the manuscript. We look forward to your decision.

On behalf of all co-authors,

Yours sincerely,

Eirlys Pijpers MSc, OT

Corresponding author

Department of Rehabilitation

Donders Institute for Brain, Cognition and Behaviour

Radboud university medical centre

PO Box 9101, 6500HB Nijmegen

The Netherlands

Email: eirlys.pijpers@radboudumc.nl

Email: recap.reval@radboudumc.nl
