# Supplementary material for: Effectiveness of the capability approach in rehabilitation for persons with neuromuscular diseases: A controlled before-after study
Source: PLoS One. 2025 Sep 23;20(9):e0332388. doi: 10.1371/journal.pone.0332388 (PMC12456807; doi:10.1371/journal.pone.0332388)
Supplement: Attachment — Submitted filename: Cover letter revision 2 response to reviewers.docx [file pone.0332388.s010.docx]

To: Tai-Heng Chen, Academic Editor PLOS ONE

Nijmegen, 20th of March 2025

Concerning: PONE-D-24-16662R1

Dear Tai-Heng Chen,

Thank you for your response regarding our submitted manuscript *Effectiveness of the capability approach in rehabilitation for persons with neuromuscular diseases: a controlled before-after study.*

We would like to thank the editor and reviewers for their constructive comments. Their feedback clearly improved our manuscript. Below, we will respond to each point addressed by the editor and reviewers and indicate where in the revised version of the manuscript our responses are implemented (line and page numbers refer to the clean copy version of the revised manuscript).

- ***Editor****: Methods section is incomplete. How N=60 participants were divided into 2 groups? Is this a RCT or not?  I noticed ref 12 was cited for more details, but manuscript to me self-contained, it should include information that minimally required for understanding the method.*

We have added extra information in the methods section (p 5, lines 90-93) to better understand the research design and method. The study is not an RCT but a controlled before and after design (see p 5 line 89); which means that the first group was included and received usual care. Next, the healthcare professionals were trained in providing capability care (intervention), and then the second group was included to receiving the intervention. Each group contained 30 participants (added on p 5 lines 90-93); the sample size calculation can be found on p 9.

- ***Editor****: Statistical analysis section says  “For an intervention effect to be present, both the COPM-P and COPM-S needed to show a significant difference between groups (p < 0.05)”. Should also the difference be more than the minimum clinically significant difference (MCID) for it to be practically important?*

For our power calculation, we have used the p-value to determine whether there was a significant difference between groups. We agree that a MCID is relevant. However, for our primary outcome measure, the COPM, there recently has been discussion in the literature about what a clinically relevant change is; which we discuss in our discussion section (p 18-19 ‘effect of rehabilitation: changes over 6 months’). From our results there is no difference between groups but both groups do show an improvement over time. However, we are not able to say whether these improvements are clinically relevant or not (which we mentioned in our discussion section).

- ***Editor****: Results for the main outcome measure was given as Pvalues (ie, statistical significance). More useful info for readers is the size of the effect (ie, difference in means with CI). Difficult to assess practical importance of observed difference when confidence intervals not presented. This info should be already available in your current analysis, why not report them? Same for table3 where mean difference is given but not the CI for that. Presenting CIs allows readers to see the observed difference relative to MCID given in sample size calculation section.*

We have added the confidence intervals in the results section on pages 13-14 lines 270-285. We have therefore added a new table (table 4) and have adapted table 3 (now table 5).

- ***Editor****: Under the secondary outcome measures in results section authors talk about “subscales” that was never mentioned in methods. What are these subscales? I didn’t find the answer even in the protocol submitted as supplementary material.*

We have removed the text ‘per subscale’ in the results section (p 14, line 287) as we agree that this text could confuse the reader. What we intended to say is that not all outcome measures have total scores and therefore the analyses are performed on each of the subscales, for example subscales of the SF-36. However, in the section ‘outcome measures’ of the methods (p 7-9) we already described which scores we use (some being total scores and some subscales).

- ***Editor****: Need a careful re-reading of the manuscript to eliminate inconsistencies. For example, EQ5D-5L-VAS is described as a measure in 0-10 scale, this is contradictory to the information in table 5 which are out of this range.*

Thank you for pointing out this inconsistency. The EQ-5D-5L-VAS has a range from 0-100. We have adapted this in the methods section (p 8 line 160).

- ***Reviewer #1:*** Elaborating/mentioning the impact of even slight cognitive impairment (only severe cognitive impairment was an exclusion criteria) on choosing activities/tasks/functionings, setting goals and conceptualizing well-being would be interesting as this might represent a limiting factor especially with your DM1 population

We agree from a clinical perspective that this might indeed impact especially the DM1 participants. However, our analyses show that there is no difference between DM1 and FSHD. Type of NMD is included as a covariate but showed no significant effect on the primary outcome measure and most of the secondary outcome measures. Type of NMD as a covariate did show a significant effect on the USER-P restrictions and satisfaction scales, on the ICECAP-A sum score and tariff value, and on the SF-36 RP (role limitations due to physical health problems) (see appendix table 10, p 24). However, it is not clear whether this can be attributed to cognitive impairment as we have not measured this. We have now added the following sentence in our discussion (p 16-17 lines 345-347): “We also did not include a measure on cognitive impairment, which might be (more) present in DM1 and might have impacted on goal setting and conceptualizing well-being.”

- ***Reviewer #1:*** *296: typo "them"*

We have adapted this (now p 15 line 316).

- ***Reviewer #1:*** *How have patients been blinded? if they did not see any difference in the care you were providing, they would know they were not in the tested group, hence they might have been disappointed, hence this would impact their well-being?*

Participants have been blinded by not telling them which care they received. We agree with your comment that if they did not experience any difference with the care they are used to that they might have a suspicion of the group they were in. To check whether participants had a suspicion of the care they received, after the T1 measurements, we asked them (over phone or videocall) whether they had a suspicion and what their reasoning for this suspicion was. In each group 4 participants mentioned that they did not experience a difference with previously experienced care. In the capability care group 6 participants (21%) mentioned reasons that could be attributed to capability care. We have added this information in more detail, including a new table (table 3), in the methods (p 5 lines 94-96) and results (p 12-13 lines 251-263) section. We consider this 21% as sufficiently low – e.g. 25% in the capability care group had no suspicion of group and 21% in this group incorrectly suspected that they received usual care; which we added in the discussion section (p17 lines 351-356).

- ***Reviewer #1:*** *In conclusion, I think that finding/investigating new approaches/concepts/frameworks to enhance rehabilitation in NMD is really important, but to me here the real difference between the rehabilitation provided following multidisciplinary care team meetings and the capability care is not crystal clear.*

We agree with the reviewer that it is important to investigate new approaches and to the best of our knowledge, we are the first to research the implementation of the capability approach in rehabilitation care. In this research project, capability care has been developed and implemented for our population and setting and during this process we have had similar discussions about the added value for our setting. The main discussions were about 1) whether the contrast between usual care and capability care is sufficiently large, as rehabilitation care already has shifted towards a broader focus on well-being using the ICF, and 2) whether only one ‘day of analysis and advice’ can bring about a substantial change in well-being by using the capability approach, or that a full rehabilitation treatment is needed (that we do not provide in our setting). These topics are mentioned in the discussion section (see ‘content of capability care’, p 17-18). A process analysis has already been started to provide us with more insights into the implementation of capability care and how this changed rehabilitation in our setting. This process analysis will be published separately.

We hope that our responses are clear and that you will accept our revision of the manuscript. We look forward to your decision.

On behalf of all co-authors,

Yours sincerely,

Eirlys Pijpers MSc, OT

Corresponding author

Department of Rehabilitation

Donders Institute for Brain, Cognition and Behaviour

Radboud university medical centre

PO Box 9101, 6500HB Nijmegen

The Netherlands

Email: eirlys.pijpers@radboudumc.nl

Email: recap.reval@radboudumc.nl
