# Supplementary material for: Effectiveness of the capability approach in rehabilitation for persons with neuromuscular diseases: A controlled before-after study
Source: PLoS One. 2025 Sep 23;20(9):e0332388. doi: 10.1371/journal.pone.0332388 (PMC12456807; doi:10.1371/journal.pone.0332388)
Supplement: Attachment — Submitted filename: Cover letter revision 3 with response to reviewers.docx [file pone.0332388.s011.docx]

To: Julie Dumonceaux, Academic Editor PLOS ONE

Nijmegen, 9^th^ of July 2025

Concerning: PONE-D-24-16662R2

Dear Julie Dumonceaux,

Thank you for your response regarding our submitted manuscript *Effectiveness of the capability approach in rehabilitation for persons with neuromuscular diseases: a controlled before-after study.*

We would like to thank the reviewers for their constructive comments. Their feedback clearly improved our manuscript. Below, we will respond to each point addressed by the reviewers and indicate where in the revised version of the manuscript our responses are implemented (line and page numbers refer to the clean copy version of the revised manuscript).

- ***Reviewer #4:*** *Interpretation of negative findings
  The manuscript now more clearly states that the study found no difference in outcomes between capability care and usual care. However, the discussion could benefit from further elaboration on what this equivalence might mean in clinical terms. For instance, does this imply that capability care is a viable alternative without additional harm or cost? Or should this outcome be seen as a limitation of the intervention as implemented?*

We agree with the reviewer that it is an interesting question what our results mean for clinical practice. The intention of our study was to develop an intervention that would improve well-being of persons with NMD compared to persons receiving usual rehabilitation care. To gain more insights in the results of our study, we have performed a process evaluation (manuscript submitted). This process evaluation showed that there were challenges with implementation, which could be one of the reasons for our results. In this separate paper we address these issues in more detail, including a discussion on what this means for clinical practice. We feel that it is beyond the scope of the current manuscript to address these issues here while the process evaluation allows room for a thorough discussion on this topic.

- ***Reviewer #4:*** *Potential ceiling effects and sensitivity
  Given that both groups improved equally on the COPM, I suggest including a short discussion on the possibility of ceiling effects or limited sensitivity of the COPM to detect differences in this specific patient population or care context.*

We have adapted the manuscript (p15-16, lines 318-320) to describe that the COPM has been used for evaluating interventions for persons with NMD and that it has been shown to have satisfactory to excellent measuring properties, including sensitivity. Considering the baseline and follow up scores in our study range between 4.4-5.0 (baseline) and 5.5-5.8 (follow-up), we do not feel that there is a ceiling effect, as the maximum score of the COPM is 10. As described above, to gain more insights into our results, including the equal improvement in both groups, a process evaluation has been performed and a separate manuscript that discusses possible reasons for this has been submitted.

- ***Reviewer #4:*** *Generalisability and context
  The intervention is described in a highly specialised Dutch tertiary care context. A short reflection on the potential challenges of implementing “capability care” in other settings (e.g., primary care, other countries) would enhance the value of the discussion.*

We agree that implementation in a variety of healthcare settings would require different implementation strategies, and probably also an adaptation of the capability care intervention itself. However, as we have performed a process evaluation that provides us with more insights into the implementation challenges in our setting, we feel that this discussion is more at it’s place in the discussion section of the process evaluation manuscript (submitted). This allows us room for a more extensive discussion on this topic. Therefore, we have not included this in the current manuscript.

- ***Reviewer #4:*** *Language and clarity
  A final language check would be beneficial to further improve fluency. For example, some sentences remain long and abstract in the Results and Discussion sections.*

We have checked our language and have adapted some sentences in the results and discussion sections, improving fluency.

- ***Reviewer #5:*** *First off, I have no objections or criticisms of the underlying scientific conduct of the study or the write up. In its own terms it is conducting research professionally and competently. Nor will I be copy editing, despite some irritating lapses (e.g. the ICF is both mistitled and mis-referenced), although I would recommend someone reviewing the paper in detail for slips like this.*

As described above we have made some adjustments to the manuscript to further improve fluency. We have added the ICF webpage as a reference; however, to the best of our knowledge we did not mistitle the ICF in our manuscript as we use the exact words of the WHO: International Classification of Disability, Functioning and Health.

*My concern (and the reason for the ‘major’ rather than ‘minor’ revision recommendation), is that it is fundamentally confused about Sen’s capability theory. Or to put it another way the primary proxy outcomes measures (COPM-P, -S) or the secondary outcome measures (although USER-P is closest) are conceptually unlinked, i.e. they are not proxies of capability at all. Ultimately, the problem is that capability is not quality of life (that is acknowledged) nor is it ICF functioning (see, e.g. https://doi.org/10.1016/j.alter.2013.08.003), It is very different. in Sen’s account it is a measure of political equality (or level of development) based on opportunities.*

We are well aware of the problem around measuring capabilities and the discussions in the literature on this topic. With the choice for the COPM we are indeed measuring functionings, and we realise that the COPM might not even be able to capture the functionings that do not directly relate to occupations. We have also included the ICECAP-A as a measure, however, as this is not an individualised measure it might not capture changes important to the person with NMD. In our discussion section we already included a discussion on this topic (pages 15-17 lines 311-362), concluding with: “the measures we have chosen might not have been able to capture a change in well-being as conceptualised in the capability approach. Currently, there is no capability well-being measure that is suitable for the context of personalized (rehabilitation) care. While methods for evaluating the impact of interventions on capabilities are still in development and challenges need to be addressed, recommendations and guidance for this development are available” thereby referring to Rijke et al. (2023) (reference 42 in manuscript).

*The authors ignore in their choice of outcome measures that capacities can only be assessed in terms of the external, physical, social, political, economic resources that the person has access to and actually uses. This is not just a matter of a person’s intrinsic functioning ability (what they can do, after rehabilitation), nor is it a matter of their satisfaction with what they can do. It is a measure of what, in their actual circumstances, they can actually do. In ICF terminology it is a measure of performance not capacity. Unfortunately, teasing apart the impact of environmental supports, resources, etc from the actual performance has proven extremely different -- for both the ICF approach and capabilities. As a result, Sen’s account has been difficult to operationalize and use in practice. From Sen’s point of view, that is not a problem since his is a contribution to political theory more than direct application, although there is some literature suggesting that Nussbaum or Venkatapuram offer capability accounts of health that are more amenable to operationalization.*

*So, the foundations of this paper are problematic. I don’t see how that can be easily fixed since it foundational.*

*Perhaps, in fairness, if the paper acknowledged that their capability approach is not Sen’s (or the others in this area) but one inspired by the insights of the capability approach (or something else as modest), then readers won’t be mislead. And indeed that would be a minor revision.*

Thank you for these interesting observations which are, in fact, reminiscent of discussions that we have had in our team. Indeed, as observed by many, the concept of capability is as inspiring and intriguing as it is difficult to operationalize. We do think that we have measured some of the elements of the capability concept, but, indeed, only some of them. We tend to think of capability as freedom, and what people in fact do and are, while making important assumptions, can be considered to be at least partially reflective of their freedom, especially when taking into account relevant resources and conversion factors. However, we feel that this paper is not the place for further discussion of this topic. We therefore gratefully adopt the suggestion to downplay our suggestion that ours would qualify as a full operationalization of the capability concept. Indeed, on a more positive note, we like the suggestion that our project (which included the development of a novel approach in rehabilitation for persons with NMD, training of the various professionals within the team, implementation and a process and outcome analysis) was *inspired* by Sen’s work on capability.

To clarify this to the readers we have adapted the manuscript on page 4 (line 62), where we deleted Sen’s name in relation to the description of the capability approach. We also changed the description of our work being ‘inspired by’ instead of ‘based on’ the capability approach in several places in the manuscript (p 2 line 26, p 3 line 43, p 5 lines 78 and 82, p 15 line 298 and 311, p 20 line 421).

We hope that our responses are clear and that you will accept our revision of the manuscript. We look forward to your decision.

On behalf of all co-authors,

Yours sincerely,

Eirlys Pijpers MSc, OT

Corresponding author

Department of Rehabilitation

Donders Institute for Brain, Cognition and Behaviour

Radboud university medical centre

PO Box 9101, 6500HB Nijmegen

The Netherlands

Email: eirlys.pijpers@radboudumc.nl

Email: recap.reval@radboudumc.nl
